# The association between frequency of eating out with overweight and obesity among children aged 6–17 in China: a National Cross-sectional Study

**DOI:** 10.1186/s12889-021-11104-0

**Published:** 2021-05-28

**Authors:** Yanning Ma, Weiyan Gong, Caicui Ding, Chao Song, Fan Yuan, Jing Fan, Ganyu Feng, Zheng Chen, Ailing Liu

**Affiliations:** Department of Nutrition and Health Education, National Institute for Nutrition and Health, Chinese Center for Disease Control and Prevention, No. 27 Nanwei Road, Xicheng District, Beijing, 100050 China

**Keywords:** China, Eating out, Frequency, Overweight and obesity, Children

## Abstract

**Background:**

In parallel with the increased prevalence of childhood overweight and obesity, the proportion of eating out in China has increased dramatically in recent years. The purpose of the study was to explore the association between frequency of eating out with overweight and obesity among Chinese children.

**Methods:**

The representative sample was recruited from Chinese National Nutrition and Health Survey (CNNHS) in 2010–2012, which included Chinese children aged 6–17 years (7685 boys and 7576 girls). Frequency of eating out was collected by interview-administered questionnaire and categorized as: 0, 1–2 and ≥ 3 times per week. Height and weight were measured, consequently body mass index was calculated.

**Results:**

The prevalence of eating out among Chinese children aged 6–17 years old was 23.2%. Children who ate out 0, 1–2, ≥3 times per week were accounted for 76.8, 10.9 and 12.3% respectively. Findings revealed that eating out three times per week or more was statistically significant associated with higher prevalence of overweight and obesity among boys (OR = 1.20,95CI:1.04–1.38) compared with those ate out less than three times per week. However, no significantly association was observed among girls (OR = 0.91, 95CI:0.78–1.01). In additional, Younger children, rural children, children from low income family, those with leisure exercises (certain physical activities regularly carried out such as swimming, walking, running, equipment fitness), leisure time sedentary behaviors (LTSB)(> 2 h/d) were relatively more likely to eat out.

**Conclusions:**

The results illustrated that eating out three times or more had a significantly positive effect on overweight and obesity among boys in China.

**Supplementary Information:**

The online version contains supplementary material available at 10.1186/s12889-021-11104-0.

## Background

The worldwide prevalence of overweight and obesity among children has becoming a global health problem [1, 2]. The prevalence of overweight and obesity among children has increased by 47.1% globally between 1980 and 2013 [3]. Increases were observed in both developed countries and developing societies [4]. The number of childhood overweight and obesity also increased dramatically over the past decades in China [5]. A recently report among Chinese students aged 7–18 years old showed that the nationwide prevalence of overweight and obesity increased from 2.1to 12.2%, 0.5to 7.3%from 1985 to 2014, respectively, and this proportion was expected to reach 28% or affected 3496 million children by 2030 [6]. The previous studies suggested that overweight and obesity children had an risk of adult obesity [7], which will result to an increased morbidity and mortality [8, 9]. Childhood overweight and obesity correlate with high blood pressure, heart disease and other chronic disease [10]. Childhood overweight and obesity is attributed to complex interaction of genetic, environmental and other behavior factors [11, 12].

In parallel with rapid economic development, the dietary structure and eating behaviors have undergone great changes [13, 14], eating out has become a continuously growing part of the Chinese dietary pattern. According to the statistics released by China‘s National Bureau of Statistics,carting revenue increased from 74.03 billion RMB in 2002 to 441.99 billion RMB in 2012, which raised almost 5 times [15]. In terms of eating out associate with higher intake of total energy, total fat, sodium, and sugar and lower intakes of fiber, vitamins, and minerals [16, 17],the available data have suggested that eating at restaurant may increase the consumption of meat, sweet while reducing the intake of grains, vegetables and fruits [13]. Furthermore, a large numbers of literature have indicated that eating out was associated with higher intake of energy [13, 16–18], the increasing energy intake from restaurant drove overall increase in daily energy [19]. thus it may contribute to energy unbalanced that cause weight gain [16]. As number of eating out occasion continues to increase, more attention are paid to the relationship between eating out with overweight and obesity.

Aristides [20] et al. demonstrated that children who ate at restaurant had a 21% higher risk of being overweight than those who did not eat at restaurant. Many abroad studies were conducted by nationally representative or large cohort data from west countries. A research [21] of 3–12 years children in 9 areas of China found the positive relationship between eating out and risk of overweight and obesity or BMI, mainly due to the substantial contribute of eating out to increase the energy intake [22]. A study [23] of Guangzhou middle school students reported that those consuming restaurant food once a week or more was associated with a 38% higher prevalence, compared with those who never ate at restaurant after adjusting for age, single child family structure, household income, and culture of students’ parents. However, available data on the association between eating out and overweight and obesity among Chinese children are far from conclusive. Besides the data of previous published was not representative of the national children population. The objective of the current study was to examine the association between frequency of eating out with overweight and obesity in a nationally representative sample of children using the data of Chinese Nutrition and Health Surveillance (CNNHS) in 2010–2012, then to provide basic information to aid in developing eating out intervention strategies.

## Method

### Study participants

Data used in this study were from the Chinese Nutrition and Health Surveillance (CNNHS) from 2010 to 2012. The nationally representative cross-sectional study was implemented by the National Institute for Nutrition and Health, Chinese Center for Disease Control and Prevention (NINH, China CDC) to assess the the nutritional status of Chinese residents. The survey covered 31 provinces, autonomous regions and municipalities (except Taiwan, Hong Kong and Macao) from 2010 to 2012. The method of multi-stage stratification and population proportional cluster random sampling was adopted. In accordance with economic development, all county-level administrative units were divided into four categories: big cities, small and medium-sized cities, ordinary rural areas and poor rural areas. A total of 150 counties (districts) were selected from four categories of areas as study sites, including 34 big cities, 41small and medium-sized cities, 45 ordinary rural areas and 30 poor rural areas, respectively. The study selected 25 households randomly from each village/community and children aged 6 to 17 years in each family were involved. If the number of children in each age group in each location is less than 20 (10 boys and 10 girls), supplementary children would be selected from nearby primary and secondary schools to reach the minimum sample size. The specific sampling method can be referred in the published literature [24]. After eliminated missing and abnormal data in height, weight, frequency of eating out and sedentary time, a total of 15,261 participants (7685 boys, 7576 girls) aged 6 to 17 years were recruited.

The informed consent was approved by the ethics review committee of the National Institute for Nutrition and Food Safety, Chinese Center for Disease Control and Prevention (No. 2013–018). All participants’ parents or legal guardian were fully informed the purpose and signed the informed consent.

### Data collection

The interview-administered questionnaire was used to collect the information of basic socio-economics, sedentary duration and leisure exercise. The food frequency questionnaire (FFQ) was used to collect eating behaviors over the past week. The details of food frequency questionnaire were attached in additional file 1. Respondents were asked “How many times have you had breakfast, lunch, or dinner in the past week (seven days)?” and “How many times in the past week have you had breakfast, lunch, or dinner at a restaurant or school canteen?”. All the questionnaires were collected by trained investigator by face-to-face interview, and the interviews were conducted at the homes of the participants. The children younger than 12 years old finished the questionnaire with the help of their parents or legal guardian. To ensure the reliability of data, quality control measures and evaluation indexes were made at the national, provincial and district levels, quality control was carried out in the period of field survey.

### Categories of frequency of eating out

In the current study, eating out was defined as eating a meal or more prepared by restaurants. Frequency of eating out was categorized as three levels: 0 time, 1–2 times, or 3 times and over per week.

### Anthropometric measurements

The height was measured with an accuracy of 0.1 cm and the fasting weight was measured with an accuracy of 0.1 kg. All measurements were conducted by well-trained investigators under standard operation procedure. Body mass index (BMI) was calculated as a division of weight in kilograms by the square of height in meters.

### Definition of weight status

Overweight and obesity was classified based on age- and gender-specific BMI cutoff points among Chinese children [25]. In the meantime, national health standard of Screening standard for malnutrition of school-age children and adolescents was devoted to screen for underweight children [26].

### Assessment of sociodemographic determinants

Age groups were divided into two categories (6–12, 13–17 years). Participants were divided into four residency groups according to location (urban, suburban, rural and poor rural). Family income was classified into four levels (< 20,000 RMB/person, 20,000–40,000 RMB/person, > 40,000 RMB/person, and unkown). Leisure exercise was defined as two levels (No/Yes). Sedentary duration status was grouped into two levels (≤2 h/d and > 2 h/d).

### Statistical analysis

All statistical analyses were conducted using SPSS 22.0. The univariate descriptive statistics were conducted for each variable (frequency and percentage), and the frequency of eating out was expressed as a percentage. Data were run to compare means between different gender, age,leisure exercise and leisure SB groups using *t* tests, data were run to compare means of region, family-income and BMI status using Chi-square tests. Multivariate logistic regression analysis was used to analyze the relationship between eating out and overweight and obesity. After adjusting for age, region, logistic regression was employed to examine the association of eating out with overweight and obesity, where dependent variable was BMI status (obesity, overweight, normal weight and underweight), and adjusted odds ratios (OR) with 95% confidence intervals (CI) were obtained. Statistical significance was considered at *p* < 0.05.

## Result

### The distribution of the characteristics among participants

The characteristics of Chinese children aged 6–17 years are presented in Table 1. In this study, the number of children involved in the survey was 15,261, 7685 boys and 7575 girls were included, and the number of urban, suburban, rural and poor rural residents was 26.1, 27.3, 33.1 and 13.5%, respectively. Children of 6–12 and 13–17 years accounted for 61.8 and 31.2%. Children from different family incomes accounted for 60.0, 15.4,4.4 and 20.2% respectively. The detailed distribution of leisure exercise, family income, leisure SB and weight status among different genders are shown in Table 1.

**Table 1:** Demographic characteristics of participant

Variables	Boys	% of Sub-Group	Girls	% of Sub-Group	All	% of Sub-Group
	N		N		N	
Age (years)						
36–12	4724	61.5	4712	62.2	9436	61.8
13–17	2961	38.5	2864	37.8	5825	38.2
Region						
Urban	1992	25.9	1995	26.3	3987	26.1
Suburban	2093	27.2	2071	27.3	4164	27.3
Rural	2566	33.4	2487	32.8	5053	33.1
Poor rural	1034	13.5	1023	13.5	2057	13.5
Family income						
Low	4628	60.2	4528	59.8	9156	60.0
Medium	1195	15.5	1153	15.2	2348	15.4
High	360	4.7	312	4.1	672	4.4
Unknown	1502	19.5	1583	20.9	3085	20.2
Leisure exercise						
No	4294	55.9	4537	59.9	8831	57.9
Yes	3391	44.1	3039	40.1	6430	42.1
Leisure SB						
≦2 h/d	2952	38.4	2928	38.6	5880	38.5
> 2 h/d	4733	61.6	4648	61.4	9381	61.5
BMI status						
Obesity	991	12.9	631	8.3	1622	10.6
Overweight	1140	14.8	875	11.5	2015	13.2
Normal	5024	65.4	5668	74.8	10,692	70.1
Underweight	530	6.9	402	5.3	932	6.1

### The frequency of eating outside of participants

The characteristics of participants were presented in Table 2. Children who ate out 0, 1–2 and ≥ 3 times per week were accounted for 76.8, 10.9 and 12.3%, respectively. Although no significant gender difference in frequency of eating out was observed, we found that there was a trend that children from economic developed region and low income family eat out more often (*P* < 0.05). Compared with their counterparts, younger children, those with leisure exercise, leisure SB (> 2 h/d) were more likely to eat out (*P* < 0.05). In additional, Table 1 shows the frequency distribution of eating out is significantly different among children with different weight status (*P* < 0.05).

**Table 2:** The frequency of eating outside of participants

Variables	Total	Eating-out (times/ week)			χ^2^	*P*
		0	1–2	≥3		
	N	N(%)	N(%)	N(%)		
Total	15,261	11,724 (76.8)	1668 (10.9)	1869 (12.3)		
Gender					3.47	0.177
Boys	7685	5887 (50.2)	822 (49.3)	976 (52.2)		
Girls	7576	5837 (49.8)	846 (50.7)	893 (47.8)		
Age (years)					98.52	< 0.001
6–12	9436	7491 (63.9)	956 (57.3)	989 (52.9)		
13–17	5825	4233 (36.1)	712 (42.7)	880 (47.1)		
Region					1127.52	< 0.001
Urban	3987	2455 (20.9)	696 (41.7)	838 (44.7)		
Suburban	4164	2996 (25.6)	551 (33.0)	617 (33.0)		
Rural	5053	4455 (38.0)	253 (15.2)	345 (18.5)		
Poor rural	2057	1818 (15.5)	168 (10.1)	71 (3.8)		
Family income					362.46	< 0.001
Low	9156	7469 (63.7)	773 (46.4)	914 (48.9)		
Medium	2348	1645 (14.0)	331 (19.8)	372 (19.9)		
High	672	386 (3.3)	147 (8.8)	139 (7.4)		
Unknown	3085	2224 (19.0)	417 (25.0)	444 (23.8)		
Leisure exercise					285.49	< 0.001
No	8831	7219 (61.6)	767 (46.0)	845 (45.2)		
Yes	6430	4505 (38.4)	901 (54.0)	1024 (54.8)		
Leisure SB					150.01	< 0.001
≤ 2 h/d	5880	4824 (41.2)	526 (31.5)	530 (28.4)		
> 2 h/d	9381	6900 (58.8)	1142 (68.5)	1339 (71.6)		
BMI status					35.34	< 0.001
Obesity	1622	1212 (10.3)	189 (11.3)	221 (11.8)		
Overweight	2015	1499 (12.8)	231 (13.9)	285 (15.3)		
Normal	10,692	8238 (70.3)	1181 (70.8)	1273 (68.1)		
Underweight	932	775 (6.6)	67 (4.0)	90 (4.8)		

### Logistic regression analysis of the relationship between frequency of eating out with overweight and obesity

The relationship between frequency of eating out with overweight and obesity were presented in Table 3. In model 1, eating out was positively and significantly associated with overweight and obesity among children (OR = 1.18, 95% CI: 1.04–1.34), No significant relationship was observed among girls (OR = 0.97,95 CI: 0.83–1.21). After further adjustment for leisure exercises and leisure time sedentary behaviors, the estimates of the association was reduced, but the significance of the association remained (model 2). Additional adjustment for age, gender, region, family income,, those who ate out 3 times or more per week had a higher risk of overweight and obesity (OR = 1.21, 95% CI: 1.03–1.41) in boys group, compared to those who ate out less than 3 times a week. No significant relationship was observed among girls (OR = 0.91,95 CI: 0.78–1.01).

**Table 3:** Logistic regression analysis of frequency of eating out and overweight and obesity.

Model	Eating out	Total		Boys		Girls	
	times/ week	OR(95%CI)	*P*	OR(95%CI)	P	OR(95%CI)	*P*
Model 1^a^							
	0	Reference					
	1–2	1.18 (1.04–1.34)	0.009	1.37 (1.17–1.62)	< 0.001	0.97 (0.80–1.18)	0.757
	≥3	1.22 (1.09–1.37)	0.001	1.37 (1.18–1.60)	< 0.001	1.00 (0.83–1.21)	0.992
Model 2^b^							
	0	Reference					
	1–2	1.13 (1.00–1.28)	0.054	1.32 (1.12–1.56)	< 0.001	0.93 (0.76–1.13)	0.471
	≥3	1.16 (1.03–1.31)	0.012	1.31 (1.13–1.53)	< 0.001	0.96 (0.79–1.16)	0.673
Model 3^c^							
	0	Reference					
	1–2	1.04 (0.92–1.18)	0.522	1.15 (0.97–1.37)	0.099	0.91 (0.74–1.11)	0.346
	≥3	1.09 (0.96–1.23)	0.178	1.21 (1.03–1.41)	0.020	0.92 (0.76–1.13)	0.432

a. model1: The model analyzed the frequency of eating out and overweight and obesity.

b. Model 2: The model adjusted leisure exercises and leisure time sedentary behaviors upon model 1.

c. Model 3: Additional adjusted for age, region, family income upon model 2.

## Discussion

In peace the rapid rapid economic development, the dietary structure and lifestyle have changed greatly. The study found that more than half of children ate out, which was higher than the national average of 28.3% as reported by the China National Health and Nutrition Survey in 2002. In parallel with an increase in the prevalence of overweight and obesity among children, the rate of eating out has increased rapidly in recent years. The current study clearly demonstrated the significant association between overweight and obesity with higher frequency of eating outside among boys, similar results were observed in previous researches: as in Brazilian children [27], and had been found among children from Etiology of Childhood Obesity (ECHO) study [28], among children and adolescents aged 11–14 years in London Borough [29], in Portugal children [20] and in US elementary schools students [30]. However, we did not observe a significant association between obesity and eating out in group of girls, which may due to boys’ greater preference for fatty food and girls pay more attention to their weight [31–33]. Whereas, the result of the UK National Diet and Nutrition Survey (NDNS) indicated that no association between energy intake and frequency of consumption of meals out in children [34]. The difference of the research results probable partly due to the lack of a unified standard for the definition of dining out. The definition for our research was refer to restaurant only, the scope is relatively limited. Moreover, at present, our research data are mainly from the results of the 2010–2012 survey, With the rapid development of catering industry in recent years. Furthermore, our research data are mainly from the finding of investigation in 2010–2012, in peace with the rapid expansion of the catering industry in recent years, consuming food from restaurant has became more convenience. Children have easy access to eat out or order online, which probably lead the increasing frequency of eating out among children. Therefore, it is necessary to take corresponding strategies and interventions to to prevent unhealthy eating behaviors among children, such as diversified health and nutrition education should be applied among children and parents.

Consistent with previous studies [18, 35], 6–12 years children were more likely to be overweight and obesity compared with 13–17 years children, which may be related to the fact that younger children tended to eat out. One possible explanation from previous research [36, 37] suggested that older children tend to choose more healthy food, which may related to the growing conception of healthy eating behavior among older children. Thus, more researches are needed to provide dietary guidance of eating out on younger children. Additionally, the study found that children from high-income household were associated with greater prevalence of overweight and obesity, the finding supported by previous researches [38, 39]. Li Miao found that children from high income family with more pocket money tend to be overweight and obesity [40]. however, opposite to previous research [31] lower rate of frequency of eating out was found among children from high-income family, which may be related to the fact that the rate of overweight and obesity was higher among children from high-income family, then they were less likely to eat out. Furthermore, The study showed that in comparison with children in rural regions, children from urban regions tended to be overweight and obesity, and this difference was also found in other domestic studies [41, 42], which was likely due to the convenience of eating out in the urban regions [43]. Similar result was founded in a survey conducted among Mexican children found that urban children [44] consumed more energy daily than those in the rural regions. Therefore, dietary nutrition education and intervention about healthy eating is necessary. As seen in the current study, children who do leisure exercise tended to eat out, It could be because of the energy expenditure caused by doing exercise, which leads children tend to foods sold in restaurants with better flavor color and taste. Leisure exercise was positive associated with high rate of overweight and obesity, which may be related to the fact that majority of childhood obesity prevention program implemented among obese rather than non-obese children [45], thus Obese children were more likely to participate in exercises [46]. We also found that children those had more leisure sedentary time tended to eat out. The similar result was found among Pakistan children aged 5 to 12 years showed that eating fast food had a significant association with sedentary behavior [47] and Matheson’s study [48]. Sedentary which included watching television and playing video game, television advertising of restaurant foods were attractive for children, which was the possible explanation of the higher rate of eating out. Preventive strategies and practical approaches may need to reduce children’s sedentary time.

The main advantages of this study were that the sample was nationally representative, which can reflect the nutrition and health status of Chinese children. The data gave a general description of eating out behavior of Chinese children aged 6–17 years, and the result provided a reference for further research with similar situation. Additionally, as eating out has become an integral part of the daily life, many researches of relationship between eating out and dietary quality among children, while, insufficient guidance of the maximum recommended frequency of eating out were existed. The result of this study provide reference times for eating out by genders. However, the current study had several limitations. Firstly, as the definition of eating out which including restaurant only. Which may underestimate the frequency of eating out; secondly, this study was cross-sectional study, the causal relationship between the eating out and overweight and obesity cannot be determined; finally, The calculated intake of energy and other nutrients is deficient, only the frequency of eating out in the interview-administered questionnaire table are analyzed. Therefore, the conclusion of the study still has practical significance.

## Conclusion

In conclusion, the result of this study demonstrated that the prevalence of eating out was 23.3% among Chinese children. Younger children, urban children and children from low-income family were more likely to eat out. Thus, dietary nutritional education and intervention about healthy eating is necessary. Considering the higher risk overweight and obesity among boys, interventions regarding eating out should tailor messages appropriately to target specific subjects by genders. From a public health perspective, the result suggested that interventions are needed to strengthen the monitoring of the situation of children eating out, thus contributing to reduce the potential enormous economic costs that are associated with eating out behavior-related illnesses, such as overweight and obesity.

## Supplementary Information

**Additional file 1.** *Food Frequency Questionnaire (FFQ)* The questionnaire was devoted in Chinese National Nutrition and Health Survey (CNNHS) in 2010–2012 to collect eating behaviors over the past week.

## Data Availability

The data sets generated and/or analyzed during the current study are not publicly available as some results are still being analyzed but are available from the corresponding author on reasonable request.

## Abbreviations

CNNHS: Chinese national nutrition and health survey
WHO: World health organization
NINH, China CDC: National institute for nutritionand health, chinese center for disease control and prevention
95% CIs: Confidence intervals
ECHO: Etiology of childhood obesity
NDNS: UK national diet and nutrition survey
LTSB: Leisure time sedentary behaviors
