# Supplementary material for: The association between frequency of eating out with overweight and obesity among children aged 6–17 in China: a National Cross-sectional Study
Source: BMC Public Health. 2021 May 28;21:1005. doi: 10.1186/s12889-021-11104-0 (PMC8161942; doi:10.1186/s12889-021-11104-0)
Supplement: Supplementary file 1 — Additional file 1. *Food Frequency Questionnaire (FFQ)* The questionnaire was devoted in Chinese National Nutrition and Health Survey (CNNHS) in 2010–2012 to collect eating behaviors over the past week. [file 12889_2021_11104_MOESM1_ESM.docx]

**Food Frequency Questionnaire (FFQ)**

The questionnaire was devoted in Chinese National Nutrition and Health Survey (CNNHS) in 2010-2012 to collect eating behaviors over the past week.

Each subject was interviewed about his/her food consumption of the past week using the food frequency questionnaire (FFQ). Participants were asked “How many days have you had breakfast,lunch, or dinner in the past week (seven days)?”,“How many days in the past week have you had breakfast, lunch, or dinner at restaurant” and subsequently “How many days in the past week have you had breakfast, lunch, or dinner at work or school canteen?”.

| F1 | The frequency of had breakfast, lunch, or dinner in the past week (seven days) | The frequency of had breakfast, lunch, or dinner at a restaurant in the past week | The frequency of had breakfast, lunch, or dinner at work or school canteen in the past week |
| --- | --- | --- | --- |
| 1.Breakfast |  |  |  |
| 2.Lunch |  |  |  |
| 3.Dinner |  |  |  |
